# Venography and Selective Ablation for Recurrent Varices after Surgery Using Radiofrequency Ablation Catheter

**DOI:** 10.1155/2021/6687450

**Published:** 2021-03-10

**Authors:** Yusuke Enta, Makoto Saigan, Akiko Tanaka, Masaki Hata, Norio Tada

**Affiliations:** ^1^Department of Cardiovascular Internal Medicine, Sendai Kousei Hospital, Sendai, Miyagi, Japan; ^2^Department of Cardiovascular Surgery, Sendai Kousei Hospital, Sendai, Miyagi, Japan

## Abstract

Recurrent varices after surgery (REVAS) is a common problem with no established treatment. Ultrasonography is a hard method to identify the source of veins that cause REVAS, especially in obese patients with thick thighs. Here, we report the case of a 64-year-old obese patient who previously underwent endothermal venous ablation for her right great saphenous vein. The patient presented with right leg swelling and venous ulceration due to REVAS. Although the source of REVAS was unclear because the patient had thick thighs on ultrasonography assessment, venography revealed that the source of REVAS was the incompetent perforator vein (IPV). Selective ablation for the IPV with radiofrequency ablation catheter was performed. We could ablate the target veins selectively so as not to ablate within the deep vein. The patient remains asymptomatic for 2 years after the procedure, and there has been no recurrence of her varicose veins. Venography allows better visualization of the source of REVAS than ultrasonography. With selective ablation, it is especially effective procedure in obese patients, in whom it is difficult to identify and access the source of REVAS with ultrasonography.

## 1. Introduction

**Figure 1:**
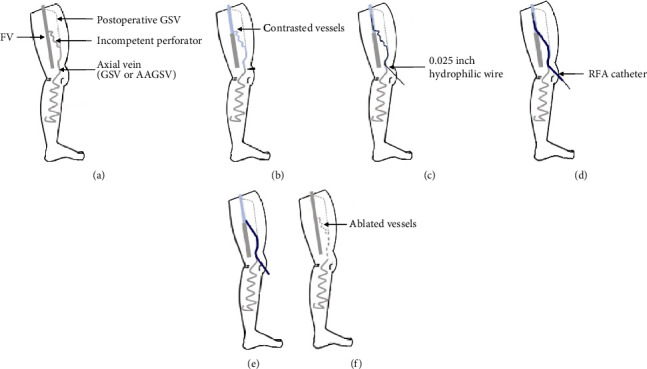
Schemas of venography and selective ablation technique. Recurrent varicose vein after great saphenous vein surgery is caused by incompetent perforator veins (IPVs) (a). Venography is performed using a 7-French sheath at axial vein around the knee (arrowhead) (b). A 0.025-inch hydrophilic wire is crossed to the femoral vein (FV) (c). A radiofrequency ablation (RFA) catheter is advanced over the 0.025-inch wire to the FV (d). The RFA catheter is pulled back to adjust the border between the FV and branch vein while contrasting from the lumen (e). Endovenous thermal ablation is performed from the IPV to the axial vein (f). GSV: great saphenous vein; AAGSV: anterior accessory great saphenous vein.

Recurrent varicose veins are known to be a common problem after surgery, recurrent varices after surgery (REVAS), in patients with chronic venous disease. The incidence of REVAS is reported to be between 20% and 80% [1–4], and no treatment has yet been established. Studies showed the REVAS to be associated with perforators, neovascularity, and recurrent saphenous insufficiency from multiple etiologies [5, 6]. It is difficult to identify the source of REVAS through ultrasonography, particularly in obese patients with thick thighs. Herein, we report the case of an obese patient with REVAS whose original veins were unclear on ultrasonography. The patient successfully underwent endovenous thermal ablation (EVTA) for target veins using the venography and selective ablation (VSA) technique with a radiofrequency ablation (RFA) catheter. The steps taken for VSA technique are presented in Figure 1. The patient with REVAS had a history of surgery for varicose veins due to great saphenous vein (GSV) insufficiency (Figure 1(a)). The patient was put in Fowler's position. We used a ClosureFAST™ (originally manufactured by VNUS Technologies Inc, California, USA, now by Medtronic, USA) RFA catheter. The RFA catheter had a lumen and could accordingly introduce the 0.025-inch wire to select and cross the target vein, advance the catheter over the wire, and flush a contrast agent from the lumen. The contrast agent was diluted 5 times with saline. We introduced the sheath to the axial vein at the knee and performed venography to identify the original veins associated with REVAS (Figure 1(b)). Thereafter, we confirmed the presence of reflux in the perforator through ultrasonography. The wire was crossed to the femoral vein (FV) via the IPVs under venography and fluoroscopy guidance (Figure 1(c)). The RFA catheter was advanced over the wire to the FV (Figure 1(d)), after which the wire was pulled out. We slowly withdrew the RFA catheter and adjusted its tip to the border between FV and the perforator vein, while contrasting from the catheter lumen (Figure 1(e)). We performed tumescent local anesthesia (TLA) under ultrasonography guidance, ablated the target vein selectively, and finally pulled the catheter and sheath out (Figure 1(f)). This procedure was performed by cardiovascular physicians at a catheterization laboratory. We performed this technique with local anesthesia and mild sedation.

## 2. Case Presentation

**Figure 2:**
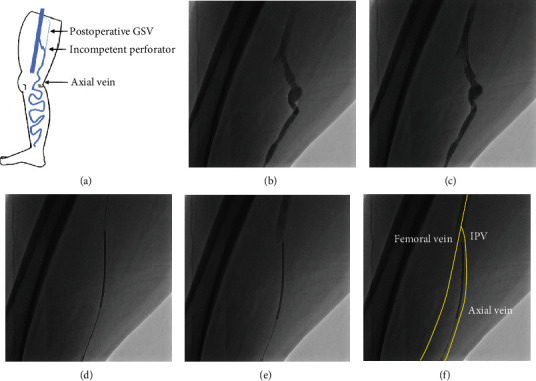
Schema of our patient's (a) recurrent varicose vein and (b–f) fluoroscopic images of venography and selective ablation technique. Venography showed a dilated incompetent perforator vein (IPV) (b). A 0.025-inch wire was crossed to the femoral vein (FV) (c). A radiofrequency ablation (RFA) catheter was advanced over the wire to the FV (d). The RFA catheter was pulled back and adjusted at the border between the FV and the perforator vein, while contrasting from the catheter lumen (e, f). GSV: great saphenous vein.

A 64-year-old female (height: 155 cm, weight: 120 kg, BMI: 50 kg/m^2^, and thigh circumference: 78 cm) with hypertension and type two diabetes mellitus presented in November 2017 with right-leg recurrent varicose veins, swelling of the ankle, pigmentation, and a history of right lower thigh venous ulceration (clinical, etiological, anatomical, and pathological clinical classification C5EpApPTPVr [7]). Her medications were a calcium blocker, an angiotensin converting enzyme inhibitor, and insulin. Previously, in 2010, she had presented with symptomatic varicose veins in the same leg. Ultrasonography had shown reflux in her right GSV without reflux in other veins. She underwent EVTA for her GSV and stab phlebectomy for the surface varicosities at another hospital. She had worn elastic stockings after first procedure, but she stopped wearing it since 2012. In 2017, she presented with a 1-year history of recurrent varicose veins in her leg. Ultrasonography showed the previously ablated GSV had atrophied. There appeared to be reflux in her axial vein at the knee, but the source of the recurrent varicose veins from the ultrasonography assessment was unclear due to her thick thighs. Therefore, we performed venography to identify the source of REVAS. Venography showed the IPV connected to her axial vein (Figures 2(a) and 2(b)), and thereafter, ultrasonography confirmed the reflux in the IPV. Therefore, we decided to ablate the IPV which caused the REVAS. We crossed a 0.025 wire from axial vein to the FV (Figure 2(c)) and advanced RFA catheter to the FV over the wire (Figure 2(d)). We withdrew the RFA catheter and adjusted its tip to the border between FV and IPV, while contrasting from the catheter lumen under fluoroscopy guidance (Figures 2(e) and 2(f)). We performed TLA and subsequent ablation from the IPV to the axial vein. The patient was discharged the day after the procedure with no deep vein thrombosis, renal damage, or any other complications. She had worn elastic stockings for a year after the procedure. After 2 years, she continued to have no symptoms, and there had been no recurrence of her varicose veins. Ultrasonography at 1-year and 2-year follow-ups showed that the ablated IPV had atrophied, and there were no deep vein thrombosis and reflux in any veins.

## 3. Discussion

Venography enabled visualizing the complex communicated veins that caused the REVAS. Selective ablation of the IPV that caused REVAS under the guidance of venography was an effective option in the case of our obese patient.

The venography allowed the complex veins communicated with REVAS to be clearly visible. A common cause of the recurrence, following the treatment of incompetent superficial veins, is IPVs. Perforator veins run near the arteries, but their anatomy is variable. This variability is more evident after significant dilatation and tortuosity caused by insufficiency, and it may render perforator veins difficult for identification through ultrasonography and hard to access by EVTA through ultrasonography assessment [8]. A previous study showed that morbid obesity (body mass index > 50) predicted the failure of perforator closure in all modalities, including USGS and EVTA [9]. It may imply that the ultrasonography images did not provide adequate assessment because the veins were obscured by the patient's thick thigh. In obese patients, using venography to diagnose the source of the REVAS offered better clarity compared with ultrasound. In absence of ultrasound experts and ultrasonography equipment, venography is a helpful alternative for diagnosing the origin of veins that cause the REVAS. Contrast computed tomography (CT) is useful for assessment of the origin of IPVs as another assessment device, but venography can visualize IPVs by fewer amount contrast agent compared to contrast CT. Moreover, procedural venography enables us to assess vessels in real time.

VSA technique has been suggested as a treatment option for REVAS caused by IPVs. IPVs play an important role in the development of chronic venous sufficiency and ulceration [10, 11]. However, redo surgery to treat REVAS caused by IPVs has not been established because it can be technically challenging, time-consuming, and is associated with a higher risk of complications [12]. As the RFA catheter has a lumen, it is possible to advance the RFA catheter toward the target veins over the wire from the lumen by venography. We can ablate the target veins selectively and not do so within the FV, because the RFA catheter is withdrawn at the same time that the veins are contrasted. The VSA technique is a less invasive procedure and takes less time than subfascial endoscopic perforator vein surgery, which is performed for perforators that cause varicose veins, and does not require the patient to undergo general anesthesia. Ultrasound-guided sclerotherapy (USGS) is another percutaneous approach for treating REVAS caused by IPVs. USGS has shown promise in perforator closure and wound healing, but with widely variable success rate [13]. Previous studies have reported that RFA was better than USGS for perforator closure and showed no neovascularization postoperatively. [14]

## 4. Conclusion

We successfully performed selective ablation for the IPV causing REVAS using an RFA catheter under venography guidance. Venography was especially effective in obese REVAS patients when assessing the structure of REVAS with ultrasonography was not possible. VSA technique should be considered a treatment option for the diagnosis and treatment of REVAS.
